# Evolutionary dynamics of ecological niche in three *Rhinogobio* fishes from the upper Yangtze River inferred from morphological traits

**DOI:** 10.1002/ece3.1386

**Published:** 2015-01-07

**Authors:** Meirong Wang, Fei Liu, Pengcheng Lin, Shaorong Yang, Huanzhang Liu

**Affiliations:** 1 Institute of Hydrobiology, Chinese Academy of SciencesWuhan, Hubei, 430072, China; 2 The Key Laboratory of Aquatic Biodiversity and Conservation of Chinese Academy of SciencesWuhan, Hubei, 430072, China; 3 University of Chinese Academy of SciencesBeijing, 100039, China; 4 China Three Gorges CorporationChengdu, Sichuan, 610041, China

**Keywords:** Ecological niche, generalist, morphological trait, niche difference, phylogenetic niche conservatism, *Rhinogobio*, specialist, the Yangtze River

## Abstract

In the past decades, it has been debated whether ecological niche should be conserved among closely related species (phylogenetic niche conservatism, PNC) or largely divergent (traditional ecological niche theory and ecological speciation) and whether niche specialist and generalist might remain in equilibrium or niche generalist could not appear. In this study, we employed morphological traits to describe ecological niche and test whether different niche dimensions exhibit disparate evolutionary patterns. We conducted our analysis on three *Rhinogobio* fish species (*R. typus*,*R. cylindricus,* and *R. ventralis*) from the upper Yangtze River, China. Among the 32 measured morphological traits except body length, PCA extracted the first four principal components with their loading scores >1.000. To find the PNC among species, Mantel tests were conducted with the Euclidean distances calculated from the four principal components (representing different niche dimensions) against the pairwise distances calculated from mitochondrial cytochrome *b* sequence variations. The results showed that the second and the third niche dimension, both related to swimming ability and behavior, exhibited phylogenetic conservatism. Further comparison on niche breadth among these three species revealed that the fourth dimension of *R. typus* showed the greatest width, indicating that this dimension exhibited niche generalism. In conclusion, our results suggested that different niche dimensions could show different evolutionary dynamic patterns: they may exhibit PNC or not, and some dimensions may evolve generalism.

## Introduction

Ecological niche describes a part of the ecological space available in the environment, which is occupied by a species (Ricklefs 2010). Ecological niche is suggested to be crucial for our understanding of the mechanism driving speciation and biological diversification, and the niche concept has become a central component in ecological research (Futuyma and Mitter 1996; Kozak and Wiens 2006; Violle et al. 2011; Gabaldón et al. 2013). In the recent decades, there have been increasing interests in analyses of how ecological niche evolves. The classical niche theory proposes that there should be distinct niche differences among species during evolution (Svensson 2012). Speciation is typically equated to divergence (Coyne and Orr 2004), and there is no maintenance of ecological similarity over time. Hence, closely related species should be morphologically and ecologically different.

In contrast, phylogenetic niche conservatism (PNC) considers that there is a tendency in species to retain ancestral ecological traits or to retain niche-related traits through speciation events over macroevolutionary time (Ackerly 2003; Wiens and Graham 2005; Losos 2008; Cooper et al. 2010; Wiens et al. 2010; Crisp and Cook 2012). Therefore, closely related species should be ecologically similar (Harvey and Pagel 1991; Ricklefs 2010; Crisp and Cook 2012). For niche differentiation, it has been suggested that, over the course of evolution, consumers may use only a narrow range of resources leading to niche shrinkage. Hence, over evolutionary time, the niche breadth should decrease and specialization of the species should increase (Ackermann and Doebeli 2004). Therefore, the generalism is not very likely in nature (Loxdale et al. 2011). However, others argue that generalists and specialists should be maintained at a balance during evolutionary process (Dennis et al. 2011). Peers et al. (2012) further suggested that specialists are presumably favored in stable or homogeneous environments, whereas generalists are likely favored in instable or heterogeneous environments. Up to date, only a few studies have been conducted to test these contrasting hypotheses.

Although the niche dimensionality hypothesis has been discussed in the past, it has received little special empirical attention (Harmon et al. 2005; Nosil and Sandoval 2008). Recently, in a study on stick insects, Nosil and Sandoval (2008) found that the degree of phenotypic and reproductive divergence between taxon pairs was positively related to the number of ecological niche dimensions: divergent selection on a single niche dimension could result in ecotype formation, while greater divergence between a species pair involved divergent selection on more niche dimensions. This is in agreement with a previous study on Caribbean *Anolis* lizards (Harmon et al. 2005) and indicates that niche dimensions might have different roles.

Most research effort on ecological niche considers analysis of trophic composition and compares spatial distribution for the target species (e.g., Sampaio et al. 2013). However, the association of a species ecological niche to its morphological characters has long been suggested and proven useful (Gatz 1979a,b, 1981; Ingram and Shurin 2009; Sampaio et al. 2013). The positive relationships between morphological variation and niche width have also been proposed (e.g. Van Valen 1965; Labropoulou and Eleftheriou 1997), especially for closely related species, despite the existence of some ambiguous results for more distantly related species. In fishes, many investigations showed that larger variations in morphological traits indicated greater niche width (Gatz 1981). Besides, Van Valen's (1965) studies on birds suggested that greater variation of morphological traits means broader ecological niche width, which later come up to a “niche variation hypothesis” (Bolnick et al. 2007; Hsu et al. 2013). Therefore, morphological data can provide useful information to infer ecological niche.

With five valid fish species, the genus *Rhinogobio* comprises medium-sized cyprinid fish endemic to the East Asian from the Gobioninae subfamily (Bănărescu 1966). All five species live on the bottom of rivers with swift current and feed on benthic invertebrates (Wu 1982). Among them, three species (*R. typus*,*R. cylindricus* and *R. ventralis*) are distributed in the upper Yangtze River and its tributaries. These three fish species are morphologically distinct, but also closely related congeners and provide a solid model to test the hypotheses related to niche evolutionary dynamics.

In this study, 33 morphological traits were measured for these three *Rhinogobio* fish species to analyze niche dimensions. The transformed morphological data of each trait were performed principle component analysis, and then Euclidean distance between species was calculated based on principal component scores to represent dissimilarity between each two species. Furthermore, the sequences of mitochondrial DNA cytochrome *b* gene of these three species were sequenced to clarify their phylogenetic relationships. Mantel tests between Euclidean distances and pairwise distances were run to find phylogenetic conservatism in different niche axes. Niche breadth in each species was estimated using the standard deviation of principal component scores, and statistical tests were performed among the different species. Our aims were to test whether (1) different niche dimensions may disparately exhibit phylogenetic conservatism or divergence; (2) some niche dimensions can become more general over evolutionary time.

## Materials and Methods

### Data preparations

**Figure 1:**
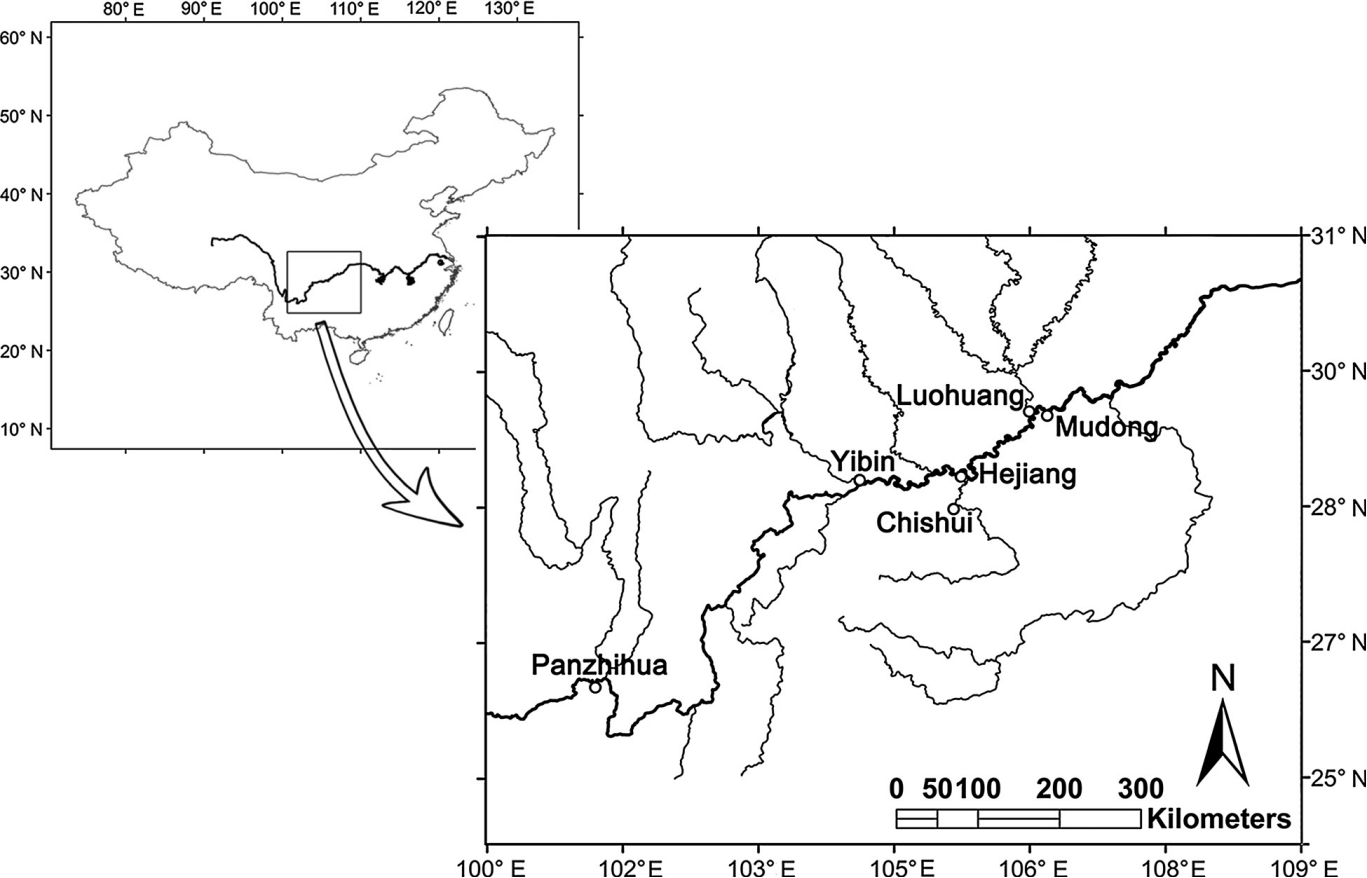
Sampling sites in upper reaches of the Yangtze River, China.

Samples of the three *Rhinogobio* fishes (*R. typus*,*R. cylindricus,* and *R. ventralis*) were collected twice from six sites in the upper Yangtze River: Panzhihua, Yibin, Hejiang, Chishui, Luohuang, and Mudong (Fig. 1). The first sampling was completed during May–June 2011; the second during September–October 2011. In total, we collected 412 specimens (Table 1). The sites where each species was collected ranged from 4 to 5, and the number of individuals of each species in one site ranged from 0 to 51. The collected samples were identified according to Wu (1982). Subsequently, after preserving 2–3 g muscle tissue from each individual in 95% alcohol, whole specimens were preserved in 8% formalin and transported to laboratory for morphological measurement.

**Table 1:** Samples employed in this study showing their numbers and localities

Sites	*R. typus*	*R. cylindricus*	*R. ventralis*
Panzhihua	0	0	36
Yibin	14	11	17
Hejiang	46	40	39
Chishui	39	0	0
Luohuang	30	51	33
Mudong	3	40	12
Total	132	142	137

**Figure 2:**
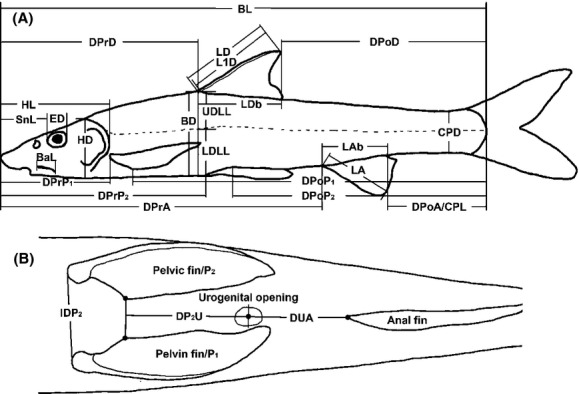
Morphometric measurements on *R. cylindricus*. (A) Lateral view from left side; (B) Ventral view. The same measurements were made on all study species.

We measured 33 morphometric traits for each specimen (Fig. 2A,B; Appendix 1 for abbreviations). All measurements were made on left side of the samples by the same person in order to minimize measurement error, and measured to the nearest 0.01 mm using digital vernier caliper, except for body length that was accurate to 1 mm. The allometric approach by Reist equation (Reist 1985) was used to remove the size-dependent variation in morphometric traits (Yang et al. 2007; Tsoumani et al. 2013). The formula of the corrected measurements was proceeded as following: *M*_*trans*_ = *log M *– *β* (*log BL *– *log BL*_*m*_), where *M*_*trans*_ was the size transformed measurement for each individual; *M* was the original unadjusted measurement; *β* was the allometric coefficient that was estimated as the slope of log *M* against log *BL*;*BL* was the body length of the individual and *BL*_*m*_ was the overall mean body length of one species while log was the base-10 logarithm. Measurements except *BL* were transformed separately using the regression slope and common overall mean body length.

After size effect removal, effectiveness of the size transformations was assessed by testing the significance of correlations between the transformed variables and *BL* (Turan 1999; Turan et al. 2005). If a significant correlation (*P *<* *0.05) is found for a transformed variable, it indicates an incomplete removal of size effects from the data (Turan 1999), and this variable is then eliminated from the analysis. However, we found no this case in our dataset. Finally, the remaining transformed data were prepared for subsequent analyses. After processing with the allometric approach by Reist equation, all the transformed data had no significant correlation with body length (*P *>* *0.05), which indicated that size effect removal was effective. The size effect removal analysis was performed using R (version 2.14.2, http://www.r-project.org/).

### Data analysis

Principal component analysis (PCA) was used to reduce the dimensionality of variables, transform interdependent variables into significant and independent components (Brosse et al. 2001), clarify the greater part of variation, and extract new composite variables (Samaee et al. 2009) interpreted the main components of the morphological variation observed as different niche dimensions. In the present work, morphological data were first transformed by Reist equation (Reist 1985) in order to satisfy the assumptions of PCA and then submitted to PCA. According to Kaiser–Meyer–Olkin (KMO) measures of sampling adequacy and Bartlett's test of sphericity (tests the null hypothesis that the original correlation matrix is an identity matrix) (Yakubu and Okunsebor 2011), the factor analysis of the transformed morphological data set was valid (*χ*^2^ = 22323.159; *P *<* *0.01). To avoid super factorization and select variables that better represent morphology, only components with eigenvalue scores greater than 1.000 were considered, following the Kaiser–Guttman criteria (Kaiser 1960). The extracted principal components (PCs) were rotated using an orthogonal rotation method (varimax) in order to simplify factors, which could help in interpreting the factors or rotated PCs. In this study, PC dimensions based on PC scores, which were computed by regression method, were interpreted as niche dimensions. The standard deviation of PC scores was used to estimate niche breadth. Euclidean distances between species of each niche dimension were estimated based on mean principal component scores. The above procedures were carried out using IBM SPSS Statistics (version 20.0, Armonk, New York, United States).

DNA extraction, PCR amplification, and sequencing of the target mtDNA cytochrome *b* gene were conducted using conventional protocols following, for example, Tang et al. (2012). Fourteen sequences of the three *Rhinogobio* fishes were chosen randomly from each sampling sites. In total five *R. typus*, four *R. cylindricus* and five *R. ventralis* were used for the analysis, with one sequence of *Coreius heterodon* from GenBank (http://www.ncbi.nlm.nih.gov/genbank/, accession number of cyt *b*: AY953000, published in Yang et al. 2006) serving as the out-group. The sequences were aligned using Clustal X (Thompson et al. 1997) and refined manually with SEAVIEW (Galtier et al. 1996). Tamura-Nei model was estimated as the best one by Modeltest 3.7 (Posada and Crandall 1998) using the Akaike information criterion (AIC) coefficients (Akaike 1973). Then, pairwise distance was calculated by MEGA 6.0 (Tamura et al. 2013) using the model. Then Mantel test (Mantel 1967), which is the common approach to study niche conservatism (Losos et al. 2003; Felizola Diniz-Filho et al. 2010) or phylogenetic signal (Pillar and Duarte 2010; Seger et al. 2013), was performed in PASSaGE (Rosenberg and Anderson 2011, http://www.passagesoftware.net/) to measure the phylogenetic effects between the two matrices, morphological niche similarity (Euclidean distances), and pairwise distances, to search for a phylogenetic conservatism in niche dimensions. The tests were carried out through the Monte Carlo randomization procedure with 1000 random permutations; the null hypothesis was that the two matrices were not related with each other at significance level of 0.01.

In the present study, we employed Bayesian approach to test for differences among different niche breadth. In WinBUGS program (version 1.4.3, http://www.mrc-bsu.cam.ac.uk/bugs), the MCMC simulations were generated, while the stationary distribution of the Markov chain was the posterior distribution of the event being investigated, from which posterior medians and credible intervals can be estimated. We employed Uniform distribution for continuous univariate, a burn-in of 10,000 iterations from the posterior distribution, to estimate posterior means, standard deviation and 95% credible intervals of niche breadth. The different values between the two group means on one niche dimension in the posterior 95% interval, which indicated that the interval did not include 0, denoted a significant difference between the two groups (*P *<* *0.05) (Suess and Trumbo 2010).

## Results

Principal component analysis produced four factors describing 83.237% of the total variance in the transformed morphological variables surveyed in this study (Table 2); the first dimension (PC1) explained 42.012%; the second dimension (PC2) explained 31.686%; the third dimension (PC3) explained 5.137%; the fourth dimension (PC4) explained 4.401%. Based on the rotated factor loading scores, we interpreted dimension 1 as a representation of niche dimension about body size variation; dimension 2 as a measure of fin length, directly represented niche dimension for swimming ability; dimension 3 as a measure of niche dimension about body height, as represented by body depth (BD), head depth (HD), and the upper distance of lateral line (UDLL); dimension 4 as a representation of niche dimension for feeding and avoiding risk, as represented by eye diameter (ED), the interdistance of eyes (IDE) and barbel length (BaL).

**Table 2:** Rotated factor loadings of morphological traits on the first four PCs from principal component analysis. Variables in bold indicate greater loading values on each dimension

Variables	PC1	PC2	PC3	PC4
% of variance	42.012	31.686	5.137	4.401
Eigenvalue	13.024	9.823	1.593	1.365
BD	−0.008	0.298	**0.848**	0.237
HL	**0.880**	−0.083	−0.095	−0.338
HD	−0.017	0.447	**0.782**	0.025
SnL	**0.821**	−0.118	−0.070	−0.384
ED	0.266	−0.517	−0.423	−**0.594**
IDE	0.633	−0.362	−0.023	−**0.571**
BaL	0.307	−0.236	−0.404	−**0.651**
LDLL	−0.260	0.257	0.597	−0.006
UDLL	−0.024	0.143	**0.861**	0.141
LD	−0.260	**0.854**	0.286	0.229
L1D	−0.202	**0.846**	0.294	0.231
LP_1_	0.044	**0.883**	0.259	−0.142
LP_2_	−0.121	**0.902**	0.254	0.153
LA	−0.234	**0.846**	0.311	0.251
LDb	0.573	0.305	0.318	0.312
LP_1_b	0.637	0.213	0.534	0.099
LP_2_b	−0.132	0.577	0.581	0.380
LAb	0.376	0.495	0.352	0.496
DPrD	**0.894**	−0.183	0.013	−0.175
DPoD	**0.913**	−0.227	−0.127	0.084
DPrP_1_	**0.887**	−0.050	−0.016	−0.293
DPoP_1_	**0.933**	−0.223	−0.046	0.057
DPrP_2_	**0.900**	−0.266	−0.034	−0.238
DPoP_2_	**0.943**	−0.182	−0.089	0.101
DPrA	**0.925**	−0.297	−0.057	−0.114
DPoA	**0.842**	0.109	−0.079	0.106
CPL	**0.843**	0.107	−0.080	0.105
CPD	0.131	0.562	0.649	0.403
IDP_1_	0.590	0.290	0.640	0.003
IDP_2_	**0.851**	−0.024	0.315	−0.055
DP_2_U	0.541	0.248	0.397	0.404

Using 14 sequences with 1140 bp from the three *Rhinogobio* species and one out-group species, phylogenetic relationships among them were analyzed, and the results were consistent with that from Wang & Liu (2005): *R*. *typus* and *R*. *cylindricus* had closer relationships than to *R. ventralis*. The pairwise distances among different species pairs were calculated to test phylogenetic niche conservatism among species. The Mantel tests between Euclidean distances and pairwise distances showed that phylogenetic relatedness and morphological niche similarity were significantly correlated for the dimensions 2 and 3 (Table 3; dimension 2: *P *=* *0.001; dimension 3: *P *=* *0.001), which indicated that the niche dimensions 2 and 3 exhibited phylogenetic conservatism among these three *Rhinogobio* species (Table 3).

**Table 3:** Mantel tests for correlations between two matrices of pairwise distances and Euclidean distances of niche dimensions. Euclidean distances based on mean principal component scores for the PC axes

Matrix 1	Matrix 2	Mantel statistic	Correlation	*P*-value
Pairwise distance	Euclidean distance for dimension 1	103.3254	0.9995	0.153
Pairwise distance	Euclidean distance for dimension 2	108.5674	−0.9978	^*^^*^0.001
Pairwise distance	Euclidean distance for dimension 3	87.7163	−0.9518	**0.001
Pairwise distance	Euclidean distance for dimension 4	97.0279	−0.1291	0.843

Significant correlations, as determined by 1000 random permutations, are indicated with asterisks

** *P *<* *0.01.

**Figure 3:**
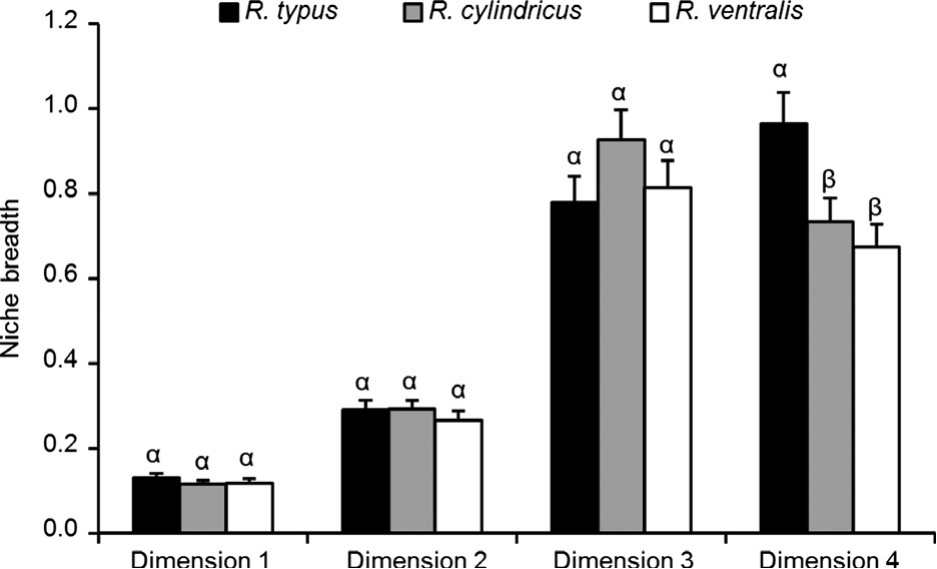
Comparison of morphological niche breadth based on standard deviation of principal component scores among the three *Rhinogobio* fishes. The bar represents the standard deviation of niche breadth. The different Greek letters indicate a significant difference among different index values (*P *<* *0.05).

Comparison of niche breadth of these three species showed that *R. typus* had the greatest width in niche dimension 4, which was significantly different (*P *<* *0.05) from *R. cylindricus* and *R. ventralis* (*P *<* *0.05) (Fig. 3). This revealed that niche breadth became more general over evolutionary time in *R. typus*. However, there were no significant differences among these three species in niche dimensions 1, 2, and 3. Considering the results of Mantel test, it showed that the relationships between niche breadth and PNC were not closely related.

## Discussion

### Morphological traits and ecological niche

Morphological properties provide the evidence for important ecological characteristics, exhibiting the strategies used by organisms and their adaptation to environment (Peres-Neto 1999; Sampaio et al. 2013), and form the basis for predictions of niche relations (Bronte et al. 1999). The inference of ecological information from morphological traits has long been established and proved useful (Hespenheide 1973; Gatz 1979a,b, 1981), as a result of recent investigations confirming strong relationships between morphological traits and ecological niche (Alfaro et al. 2005; Ingram and Shurin 2009; Sampaio et al. 2013). Using morphological information, dietary niche, and distribution data from rockfish assemblages, Ingram and Shurin (2009) showed that gill raker morphology was related to trophic position while relative eye size was associated with depth habitat. Sampaio et al. (2013) used Mantel test to investigate relationships among morphology, diet, and spatial distribution of *Satanoperca pappaterra* and *Crenicichla britskii* and found that the relationship between morphology and use of spatial and feeding resource was supported and that analyses incorporating morphological variations could contribute to our great understanding of the ecological structure of fish assemblages by providing indices on the niche characteristics of each species. Therefore, morphological analyses performed with species are useful for inferring ecological functions and exploring trophic and spatial niche (Delariva and Agostinho 2001; Abramov and Puzachenko 2012; Sampaio et al. 2013).

Furthermore, Van Valen's (1965) study on birds suggests that greater variation of morphological traits is often associated with broader ecological niche width, that is the niche variation hypothesis (NVH), which has long been debated (e.g., Grant 1967, 1979; Soulé and Stewart 1970; Dennison and Baker 1991; Meiri et al. 2005). Recently, there is increasing evidence for “niche variation hypothesis” (e.g., Simberloff et al. 2000; Blondel et al. 2002; Bolnick et al. 2007; Hsu et al. 2013). Therefore, in this study, we employed morphological traits with high factor loading values to represent ecological niche dimensions and then analyzed ecological niche dynamics.

### Phylogenetic niche conservatism and niche divergence

While there are broad theoretical foundations and empirical support of the PNC (Harvey and Pagel 1991; Ackerly 2003; Wiens and Graham 2005; Losos 2008; Cooper et al. 2010; Wiens et al. 2010; Crisp and Cook 2012; Soberón and Martínez-Gordillo 2012), other evidence rather supports niche divergence (Herrel et al. 1999; Coyne and Orr 2004; Moreno-Letelier et al. 2013). As outlined by Wiens and Graham (2005), simply testing whether niches are conserved is not by itself particularly helpful and a more useful approach should focus on the patterns that conservatism may create. In this study, by the Mantel tests between Euclidean distances and pairwise distances for the four niche axes (Table 3), we did find evidence that different niche dimensions may actually exhibit very different patterns.

In this study, dimensions 2 and 3 were found showing phylogenetic niche conservatism. It is interesting to note that these morphological traits in dimensions 2 and 3 are mainly related to fish swimming ability and behavior, which indicates that, in *Rhinogobio* fish species, the niche dimensions representing fish swimming ability and behavior are phylogenetic constrained. Therefore, in *Rhinogobio* species, different dimension of species niche could exhibit phylogenetic niche conservatism or not, and it is important to identify different dimensions while investigating ecological niches.

*Rhinogobio* species specifically live on the bottom of rivers with swift current and feed on benthic invertebrates (Wu 1982). Therefore, adaptations to fast current and benthic habitat are important to this group. Characters related to these adaptations should be much conserved. In this study, morphological traits in dimensions 2 and 3 are mainly related to fish swimming ability and behavior, adapting to the life in benthic, fast current. They were found showing phylogenetic niche conservatism. In contrast, morphological traits in dimensions 1 and 4 are mainly related to feeding mode, which were found much flexible compared with their phylogenetic relationships.

### Niche generalism and specialism

The niche evolutionary process was ever described as species dispersal, specialization, and local adaptation. Along with this process, species became more and more specialized, and the niche width becomes steadily narrower. Loxdale et al. (2011) even declared that generalism in nature is simply improbable. However, Dennis et al. (2011) argued that generalist in niche breadth not only exists, but also forms a crucial part in the evolution of specialists, and during speciation generalists and specialists may be actually maintained in a balance. Peers et al. (2012) further suggested that specialists are presumably favored in stable or homogeneous environment, whereas generalists are likely favored in instable or heterogeneous environment.

In this study, it was found that *R. typus* demonstrated significantly greatest width in niche dimension 4 (Fig. 3). This indicates that *R. typus* has become generalist in this niche dimension. Hence, niche generalism should have great foundations in the *Rhinogobio* species. It is interesting to note that Mantel tests show that dimension 4 is non-PNC dimension, which may indicate that non-PNC dimension is less phylogenetic constrained and is more likely to become generalist. However, for dimension 3 (PNC dimension), one species showed the greatest width (not significant); for dimension 1 (PNC dimension) and dimension 2 (non-PNC dimension), no one species showed the greatest width. Therefore, a clear relationship between PNC and niche width could not be established and needs some further investigations.

One notable point is that there are indeed different opinions on the “niche variation hypothesis”. However, in this study, our purpose was to address that ecological niche should be investigated at different dimensions. Different methodological opinions should not affect the identification of different niche dimensions. On the contrary, identifying different niche dimensions should be helpful for testing the “niche variation hypothesis”. Therefore, we hope that our work will stimulate further investigations in this field.

## Appendix 1: Abbreviations and biological interpretations of the 33 morphological traits employed in the present analysis

*BaL* – Barbel length. Associated with detecting prey items for benthic feeder (Gatz 1981; Kasumyan 2002); long barbel is easier to sense prey

*BD* – Body depth. Associated with swimming behavior; deeper mid-body depth is good for maneuvering and acceleration in lotic water (Walker 1997)

*BL* – Body length. Associated with prey size (Gatz 1981); larger fish have the potential of taking larger prey (Wootton and Wootton 1984)

*CPL* – Caudal peduncle length. Associated with swimming behavior; long caudal peduncle is beneficial to swimming more efficiently in rapid water flow and in general, owing the need for propulsion at short distances (Watson and Balon 1984; Domenici and Blake 1997; Breda et al. 2005; Sampaio et al. 2013

*CPD* – Caudal peduncle depth. Associated with swimming behavior; fish with a deeper caudal peduncle has a greater maneuverability potential (Winemiller 1991; Sampaio et al. 2013)

*DPrA* – Pre-anal fin distance. Associated with swimming behavior; larger value indicates higher maneuverability capacity to movement stabilization (Breda et al. 2005)

*DPoA* – Postanal fin distance. Associated with swimming behavior; larger value indicates higher maneuverability capacity to movement stabilization (Breda et al. 2005)

*DPrD* – Predorsal fin distance. Associated with the capacity to stabilization, braking in acceleration and steering abilities (Gatz 1981; Breda et al. 2005)

*DPoD* – Postdorsal fin distance. Associated with the capacity to stabilization, braking in acceleration and steering abilities (Gatz 1981; Breda et al. 2005)

*DPrP*_*1*_ – Prepectoral fin distance. P_1_ is an abbreviation for pectoral fin. Associated with maintaining the position amidst a strong current flow (Casatti and Castro 1998; Sampaio et al. 2013)

*DPoP*_*1*_ – Postpectoral fin distance. Associated with maintaining the position amidst a strong current flow (Casatti and Castro 1998; Sampaio et al. 2013)

*DPrP*_*2*_ – Prepelvic fin distance. P_2_ is an abbreviation for pelvic fin. Associated with braking (Gatz 1979a) and maintaining the position in a strong current flow (Casatti and Castro 1998)

*DPoP*_*2*_ – Postpelvic fin distance. Associated with braking (Gatz 1979a) and maintaining the position in a strong current flow (Casatti and Castro 1998)

*DP*_*2*_*U* – Pelvic-fin to urogenital opening distance. Associated with reproductive behavior

*DUA* – Urogenital opening to anal fin distance. Associated with reproductive behavior

*ED* – Eye diameter. Associated with visual sensitivity (Sibbing and Nagelkerke 2001) and foraging position in the water column (Gatz 1979a; Fieire and Agostinho 2001); species that inhabit deeper areas have relatively smaller eyes (Gatz 1979a; Wikramanayake 1990

*HD* – Head depth. Associated with food size (Oliveira et al. 2010; Sampaio et al. 2013); larger head is considered as an adaptation for larger prey items (Gatz 1979a, 1981)

*HL* – Head length. Associated with food size (Oliveira et al. 2010; Sampaio et al. 2013); larger head is an adaptation for larger prey items (Gatz 1979a, 1981)

*IDE* – Interdistance of eyes. Possibly associated with the visual range of fish underwater; visual range is crucial for feeding opportunities and avoiding risk (Nicol and Somiya 1989)

*IDP*_*1*_ – Interdistance of left and right pectoral fin. Associated with the capacity of grasp and promoting station-holding opposing water current for benthic fishes (Arnold et al. 1991). This lateral position of pectoral fins may enhance yaw maneuvering relative to fish with ventrolateral fins (Drucker and Lauder 2002; Lauder and Drucker 2004)

*IDP*_*2*_ – Interdistance of left and right pelvic fin. Associated with the capacity of grasp and promoting station-holding opposing water current for benthic fishes (Arnold et al. 1991); large value may enhance yaw maneuvering

*LA* – Anal fin length. Associated with maneuverability capacity and movement stabilization (Breda et al. 2005; Standen and Lauder 2005)

*LAb* – Anal fin base length. Associated with maneuverability capacity and movement stabilization (Breda et al. 2005; Standen and Lauder 2005); large fin base increases the control force of muscles that control both fin position relative to fish body as well as surface conformation, allowing fish to alter fin shape during locomotion (Lauder and Drucker 2004)

*LD* – Dorsal fin length. Associated with capacity to stabilization and braking in acceleration (Breda et al. 2005)

*LDb* – Dorsal fin base length. Associated with maneuverability capacity and movement stabilization; large fin base increases the control force of muscles that control both fin position relative to fish body as well as surface conformation, allowing fish to alter fin shape during locomotion (Lauder and Drucker 2004)

*L1D* – The first branched dorsal fin ray length. Associated with capacity to stabilization and braking in acceleration (Breda et al. 2005)

*LP*_*1*_ – Pectoral fin length. Associated with swimming performance and maneuvering (Lauder and Drucker 2004); longer pectoral fins are beneficial for maintenance of the position in a strong current flow for benthic fish inhabiting rapids (Casatti and Castro 1998

*LP*_*1*_*b* – Pectoral fin base length. Associated with locomotion; large fin base increases the control force of muscles that control both fin position relative to fish body as well as surface conformation, allowing fish to alter fin shape during locomotion (Lauder and Drucker 2004), and maintain fairly horizontal body positions during steady forward swimming (Rosenberger 2001)

*LP*_*2*_ – Pelvic fin length. Associated with locomotion; longer pelvic fins are benefit for maintenance of the position in a strong current flow for benthic fish inhabiting rapids (Casatti and Castro 1998)

*LP*_*2*_*b* – Pelvic fin base length. Associated with locomotion; large fin base increases the control force of muscles that control both fin position relative to fish body as well as surface conformation, allowing fish to alter fin shape during locomotion (Lauder and Drucker 2004)

*LDLL* – Lower distance of lateral line. Associated with the ability to detect movements and vibrations underwater (Dijkgraaf 1963)

*SnL* – Snout length. Associated with locating prey items for benthic feeder (Gatz 1981)

*UDLL* – Upper distance of lateral line. Associated with the ability to detect movements and vibrations underwater (Dijkgraaf 1963)
